# The role of optical coherence tomography in Alzheimer’s disease

**DOI:** 10.1186/s40942-016-0049-4

**Published:** 2016-10-17

**Authors:** Leonardo Provetti Cunha, Ana Laura Maciel Almeida, Luciana Virgínia Ferreira Costa-Cunha, Carolina Ferreira Costa, Mário L. R. Monteiro

**Affiliations:** 1 Department of Ophthalmology, School of Medicine, Juiz de Fora Eye Hospital, Federal University of Juiz de Fora, Av. Barão do Rio Branco, 4051, Bom Pastor, Juiz de Fora, MG 36021-630 Brazil; 2 Juiz de Fora Eye Hospital, Juiz de Fora, MG Brazil; 3 Department of Neurology, School of Medicine, Federal University of Juiz de Fora, Juiz de Fora, Brazil; 4 Division of Ophthalmology, University of São Paulo Medical School, São Paulo, Brazil

**Keywords:** Alzheimer’s disease, Mild cognitive impairment, Dementia, Optical coherence tomography, Macula, Retina, Optic nerve, Retinal nerve fiber layer, Ganglion cell layer

## Abstract

**Background:**

Alzheimer’s disease (AD) is the most common cause of dementia and its incidence is increasing worldwide along with population aging. Previous clinical and histologic studies suggest that the neurodegenerative process, which affects the brain, may also affect the retina of AD patients.

**Main body:**

Optical coherence tomography (OCT) is a non-invasive technology that acquires cross-sectional images of retinal structures allowing neural fundus integrity assessment. Several previous studies demonstrated that both peripapillary retinal nerve fiber layer and macular thickness measurements assessed by OCT were able to detect neuronal loss in AD. Moreover, recent advances in OCT technology, have allowed substantial enhancement in ultrastructural evaluation of the macula, enabling the assessment not only of full-thickness retinal measurements but also of inner retinal layers, which seems to be a promising approach, mainly regarding the assessment of retinal ganglion cell layer impairment in AD patients. Furthermore, retinal neuronal loss seems to correlate with cognitive impairment in AD, reinforcing the promising role of OCT in the clinical evaluation of these patients.

**Conclusion:**

The purpose of this article is to review the main findings on OCT in AD patients, to discuss the role of this important diagnostic tool in these patients and how OCT technology may be useful in understanding morphological retinal changes in AD.

## Background

Alzheimer’s disease (AD) is the most common cause of dementia and its incidence is increasing worldwide associated with population aging [1]. AD is characterized by progressive cognitive impairment, such as memory deficit, decline in learning and executive functioning, aphasia, apraxia, agnosia and visual abnormalities [2, 3].

Visual complaints are common findings in AD patients and these may have an important impact on autonomy and quality of life of these patients. The most common visual symptoms are impairment of spatial contrast sensitivity, motion perception, color discrimination and visual loss, which in the past, were attributed to lesions affecting the primary visual cortex and other specific areas of the brain [3–6]. Neuroimaging techniques are essential in the diagnosis of AD and magnetic resonance imaging (MRI) has become the most used tool for cerebral imaging in AD patients, providing detailed information about brain structure. The most common findings in MRI of patients with AD are atrophy in the medial temporal lobe, including hippocampus, amygdala, entorhinal cortex and parahippocampal gyrus, ventricular enlargement and reduction of total brain volume [7]. Although studies have not yet completely elucidated the structural and functional changes that occur in brains of AD patients, some clinical and histologic studies suggest that the same neurodegenerative process that occurs in the brain, may also affect the retina, since the latter represents a peripheral part of central nervous system. Retinal pathological changes such as loss of retinal ganglion cell (RGC) and their axons were demonstrated, both in animal models and in *post mortem* studies of human AD eyes [8–11]. Toxic aminoacids, such as fibrillar tau and Aβ aggregates were accumulated within the retina and its microvasculature, and signs of neuroinflammation were present in the retina [12–16]. Therefore, according to several clinical and histologic studies there is strong evidence of anterior visual pathway impairment in AD patients, with predominant involvement of RGC and their fibers [10, 11, 17–19].

Optical coherence tomography (OCT) is a non-invasive technology, which acquires cross-sectional images of retinal structures allowing neural fundus integrity assessment. Over the last years, OCT became the most widely used technology to detect and quantify structural axonal damage in many optic nerve and neurological diseases. Axonal loss is usually quantified by measuring OCT peripapillary retinal nerve fiber layer (RNFL) that allows an indirect estimation of RGC layer impairment. Furthermore, neuronal loss can be directly accessed by estimating macular thickness measurements, since 30–35 % of the retina thickness in macular area is composed by the RGCs and their fibers, as previously demonstrated in eyes with glaucoma, papilledema, compressive or demyelinating optic neuropathies [20–22]. If we take into account that the retina is considered a peripheral extension of the brain and both share similar embryological origin, it is easy to understand why OCT has become a widespread diagnostic tool in many neurological diseases.

Therefore, the purpose of this article is to address the main findings on OCT in AD patients, to discuss the role of this important diagnostic tool in these patients and how OCT technology could be useful to understand morphological retinal changes in AD.

## Peripapillary retinal nerve fiber layer thickness in Alzheimer’s disease patients

The degeneration of the optic nerve and consequent loss of ganglion cells and their axons was first demonstrated histologically in patients with AD around 30 years ago [10]. Other studies confirmed these findings, revealing a predominant loss of the largest RGCs (M-cells) [11]. These *post*-*mortem* studies are clear evidence of anterior visual pathway impairment in AD patients. Hedges et al. [23] evaluated fundus photographs from 26 AD patients and found a high incidence of RNFL abnormalities.

With the advent of OCT, over the last two decades, it became possible to provide a directly clinical quantitative assessment of retinal axonal loss. Several previous studies have evaluated the peripapillary RNFL thickness measurements assessed by OCT and all of them were able to demonstrate that most of RNFL parameters were reduced in patients with AD [19, 24–34]. The reduction of RNFL thickness was significantly greater than that which is observed in the age-matched controls and thus cannot be exclusively ascribed to aging. In accordance with these studies, the reduction of RFNL thickness occurred in each of the four retinal quadrants, suggest that axonal loss in patients with AD seems to be the result of a diffuse degeneration process of RGCs. Global reduction of peripapillary RNFL average thickness measurements in AD patients was demonstrated by several independent groups [24, 28, 30, 31, 34]. Most of them observed a significant reduction of RNFL thickness in all quadrants [19, 24, 31, 35, 36], with a predominance in the superior [26–30] and inferior quadrants [24, 27, 30]. Different groups showed the axonal loss with RNFL reduction in AD patients, despite different commercially available OCT devices used [37, 38].

Thomson et al. [39] conducted a systematic review and meta-analysis of the literature to determine the diagnostic utility of OCT of the RNFL thickness measurements in various dementias, but focused predominantly in patients with AD and mild cognitive impairment (MCI). They included studies published until September 2014. They identified a total of 17 studies including 702 AD eyes and 790 control eyes. There was a significant reduction in the overall, inferior and temporal RNFL thickness measurements in AD patients compared with controls, regardless of whether time domain (TD) or spectral domain (SD) OCT was used. The nasal and temporal quadrants are only found to be significantly thinner in few studies. These authors concluded that studies analyzed in the systematic review suggest that the significant thinning of the RNFL does occur in AD, and that OCT can be successfully used to detect these changes.

In our review, we identified a total of 23 studies including 1330 AD eyes, 326 MCI eyes and 1082 control eyes. In nine studies TD-OCT was used and in the other fourteen, the patients were evaluated by SD-OCT. Seven studies included MCI [25, 28, 34, 35, 40–42] patients and others nine evaluated macular parameters [24–26, 31, 35, 40, 43–45]. Table 1 summarizes the demographic data and RNFL thickness measurements in Alzheimer’s disease, MCI and controls by OCT.

**Table 1:** Demographic data and retinal nerve fiber layer thickness measurements in Alzheimer’s disease, mild cognitive impairment and controls by OCT

Study	OCT type	Diagnosis, number of subjects (eyes)	Mean age ± SD (years)	Mean MMSE ± SD	Mean peripapillary RNFL SD (µm)	Notes
Parisi et al. [19]	TD	AD, 17 (17)	70.4 ± 6.1	16.4 ± 2.4	59.5 ± 16.8**	The mean peripapillary RNFL thicknes correlated with PERG
		Controls, 14 (14)	Age-matched		99.9 ± 8.95	
Iseri et al. [26]	TD	AD, 14 (28)	70.1 ± 9.7	18.5 ± 6.3	87.5 ± 23.8***	The peripapillary and macular RNFL thickness of AD patients were thinner than in control subjects. Total macular volume and MMSE scores were significantly correlated
		Controls, 14 (14)	65.1 ± 9.8		113.2 ± 6.7	
Berisha et al. [46]	TD	AD, 9 (9)	74.3 ± 3.3	23.8 ± 5.1	85.5 ± 7.4	Narrow veins and decreased retinal blood flow in these veins
		Controls, 8 (8)	74.3 ± 5.8		93.8 ± 10.4	
Paquet et al. [34]	TD	AD, 26 (52)	78.5 ± 4.9		83.4 ± 7.2**	Early involvement of the RNFL in patients with MCI
		Mild AD, 14 (28)		22.6		
		Severe AD, 12 (24)		16.6		
		MCI, 23 (46)	78.7 ± 5.1	28.8	89.3 ± 2.7**	
		Controls, 15 (30)	75.5 ± 5.1		102.2 ± 1.8	
Lu et al. [30]	TD	AD, 22 (44)	73.0 ± 8.0		90.0 ± 18.0*	The RNFL thickness reductions of predominantly in the superior and inferior quadrants
		Controls, 22 (44)	68.0 ± 9.0		98.0 ± 12.0	
Kesler et al. [27]	TD	AD, 30 (52)	73.7 ± 9.9	23.6 ± 4.3	84.7 ± 10.6*	No correlation between RNFL thickness measurements and MMSE in AD patients
		MCI, 24 (40)	71.0 ± 10.0	28.1 ± 2.1	85.8 10.0*	
		Controls, 24 (38)	70.9 ± 9.2		94.3 ± 11.3	
Moschos et al. [33]	TD	AD, 30 (60)	71.8 ± 8.6			There is a functional abnormality of the outer retina in central macular area in mild stages of AD
		Controls, 30 (60)	Age-matched			
Moreno-Ramos et al. [32]	SD	AD, 10 (20)	73.0 ± 6.5	16.4	94.5 ± 2.2*	The RNFL thickness correlated significantly with both the MMSE and the Mattis Dementia Rating Scale scores in AD patients
		Controls, 10 (20)	70.0 ± 2.0		108.0 ± 2.2	
Marziani et al. [31]	SD	AD, 21 (21)	79.3 ± 5.7	19.9 ± 3.1		Macular RNFL and RNFL + GCL thickness measurements are reduced in AD patients compared with healthy subjects
		Controls, 21 (21)	77.0 ± 4.2			
Kirbas et al. [28]	SD	AD, 40 (80)	69.3 ± 4.9	21.4	65.0 ± 6.2*	No correlation between OCT parameters and MMSE
		Controls, 40 (80)	68.9 ± 5.1		75.0 ± 3.8	
Larrosa et al. [47]	SD	AD, 151 (151)	75.3	18.3	97.5 ± 14.1	Used two different OCT (cirrus and spectralis)
		Controls, 61 (61)	74.9		100.6 ± 13	
Ascaso et al. [35]	TD	AD, 18 (36)	72.1 ± 8.7 (AD + aMCI)	19.3 (AD + aMCI)	64.7 ± 15.2	The increased thickness and macular volume in aMCI
		aMCI, 21 (42)	72.1 ± 8.7 (AD + aMCI)	19.3 (AD + aMCI)	86.7 ± 7.18***	
		Controls, 41 (82)	72.9		103.1 ± 8.04	
Polo et al. [45]	SD	AD, 75 (75)	74.1	16.0	97.4 ± 11.2 (cirrus); 98.1 ± 10.7 (spectralis)	SD-OCT protocols were able to detect RNFL and macular atrophy in AD patients
		Controls, 75 (75)	73.9		99.2 ± 9.9 (cirrus); 101.6 ± 9.5 (spectralis)	
Kromer et al. [29]	SD	AD, 22 (42)	75.9 ± 6.1	22.6 ± 5.5	104.3 ± 17.5	AD patients with mild to moderate stages of showed a significant reduction of RNFL thickness in the nasal superior sector
		Controls, 22 (42)	64.0 ± 8.2		101.8 ± 10.7	
Bambo et al. [48]	SD	AD, 56 (56)	74.0 ± 8.1	16.6	89.4 ± 10.4**	Presence of optic disc pallor correlate with axonal loss and perfusion alterations in AD
		Controls, 56 (56)	76.4 ± 8.4		100.9 ± 11.7	
Bayhan et al. [43]	SD	AD, 31 (31)	75.8 ± 6.5	17.4 ± 4.9		A significant correlation with the macular GCC parameters and MMSE scores in AD patients
		Controls, 30 (30)	74.9 ± 7.6			
Liu et al. [41]	TD	AD, 67 (134)				The RNFL thickness in the superior quadrant and total mean values are gradually and significantly decreased from MCI to severe AD
		Mild AD, 24	71.3 ± 4.9		91.6 ± 10.1*	
		Moderate AD, 24	70.8 ± 6		91.7 ± 12.4*	
		Severe AD, 19	72.1 ± 4.6		87.1 ± 17.1***	
		MCI, 26 (52)	70.2 ± 6.5		95.4 ± 17.1	
		Controls, 39 (78)	69.7 ± 7.8		100.1 ± 15	
Gao et al. [25]	SD	AD, 25 (50)	74.7 ± 1.3	19.2 ± 0.6	86 ± 1.9**	Reduced macular volume in AD and MCI patients, no correlation between MMSE and OCT parameters
		aMCI, 25 (50)	73.4 ± 1.5	25.8 ± 0.35	92.4 ± 1.9*	
		Controls, 21 (42)	72.1 ± 1		98.6 ± 1.7	
Oktem et al. [49]	SD	AD, 35 (70)	75.4 ± 6.9	18.0	80.6 ± 9.6***	RNFL thickness measurements can be useful for early diagnosis and evaluation of disease progression
		MCI, 35 (70)	74.1 ± 6.3	28.0	82.5 ± 7.3	
		Controls, 35 (70)	70.2 ± 8.0	29.0	91.5 ± 7.1	
Salobrar-Garcia et al. [50]	SD	AD, 23 (23)	79.3 ± 4.6	23.3 ± 3.1		Increase in peripapillary thickness in mild-AD patients
		Controls, 28 (28)	72.3 ± 5.1			
Cunha et al. [24]	SD	AD, 24 (45)	74.8 ± 6.2	17.0 ± 5.2	93.7 ± 13.4	Neuronal loss, especially for macular parameters, correlated well with cognitive impairment in AD
		Controls, 24 (48)	72.3 ± 7.3		103 ± 9.2	
Garcia-Martin et al. [51]	SD	AD, 150 (150)	75.33	18.35 ± 3.33	95.7 ± 15.22	Performed segmentation of all retinal layers. Inner retinal layers reduction may predict greater disease severity
		Controls, 75 (75)	74.79		99.23 ± 16.48	
Choi et al. [40]	SD	AD, 42 (42)	76.8 ± 8.7	14.5 ± 5.5		Performed segmentation of all retinal layers
		MCI, 26 (26)	74.7 ± 7.8	23.1 ± 4.6	86.6 ± 10.2	
		Controls, 66 (66)	73.8 ± 7.5			

*AD* Alzheimer’s disease, *MCI* mild cognitive impairment, *aMCI* amnestic mild cognitive impairment, *RNFL* retinal nerve fiber layer, *OCT* optical coherence tomography, *SD* standard deviation, *TD* time-domain, *SD* (*OCT type column*) spectral domain, *MMSE* mini mental state examination, *PERG* pattern-reversal electroretinogram, *GCL* ganglion cell layer, *GCC* ganglion cell complex

* *P* < 0.05; ** *P* < 0.01; *** *P* < 0.001 when compared to controls

## Macular thickness measurements in Alzheimer’s disease patients

Since the RGCs layer and their axons contribute with approximately one-third of the total retinal thickness in macular area, macular thickness measurements assessed by OCT can be used to investigate neuronal loss in AD patients. This approach in AD evaluation is promising, first because RGC’s and their fibers are located in macular area, which in accordance with previous histopathological studies, are preferably affected in AD [8, 9, 17], and secondly, because of the significant improvements occurred in the latest OCT’s equipments, evolving from time-domain (TD) to spectral fourier domain technology [52]. These improvements on OCT technology provide three-dimensional high-quality images with greater resolution, with an axial resolution up to five times higher and imaging speeds approximately 60 times greater than earlier TD OCT [53, 54]. This high-density raster retinal tissue scanning allow to detect and segment the retinal structures in each raster OCT image and use these data to construct a detailed macular map, with separate analysis of different retinal layers [52].

### Full thickness macular measurements

Iseri et al. [26] were the first group to assess both macular thickness and volume by TD-OCT of 28 eyes of 14 patients with AD. The retinal thickness in all macular quadrants of AD patients was lower than in control subjects and reached statistical significance in the nasal, temporal and inferior quadrants, as well to the mean total macular volume. Moschos et al. [33] also evaluated the macular parameters using TD-OCT in AD patients. They demonstrated a preferential involvement of the central macular area.

In SD-OCT era, Polo et al. [45] evaluated macular area by SD-OCT of 75 AD patients and 75 age-matched healthy subjects. Retinal thinning was observed in AD eyes in all areas except from the fovea. In other study, Gao et al. [25] evaluated 25 patients with AD and found a significant reduction of macular volume in these patients. Salobrar-Garcia et al. [50] also demonstrated a preferential reduction of macular parameters in mild-AD patients. In this study, the peripapilary RNFL thickness was thinner, but did not reach a significant difference compared with age-matched controls, suggesting a preferential involvement of the macula in early stages of the disease.

**Fig. 1:**
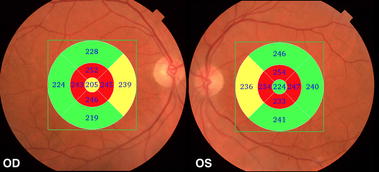
Examples of Topcon 3D OCT-2000 generated full-thickness macular measurements in the *right* (*OD*) and *left* (*OS*) eye of a patient with Alzheimer’s disease. Measurements in different sectors are indicated with *numbers* and represented in *colors* that correspond to the normal distribution. Sectors in *green* indicate values within normal range; in *yellow* less than the 5th, in *red* less than the 1st compared with an age-matched reference population. Note that several macular thickness parameters are below normal range, particularly in inner segments

**Fig. 2:**
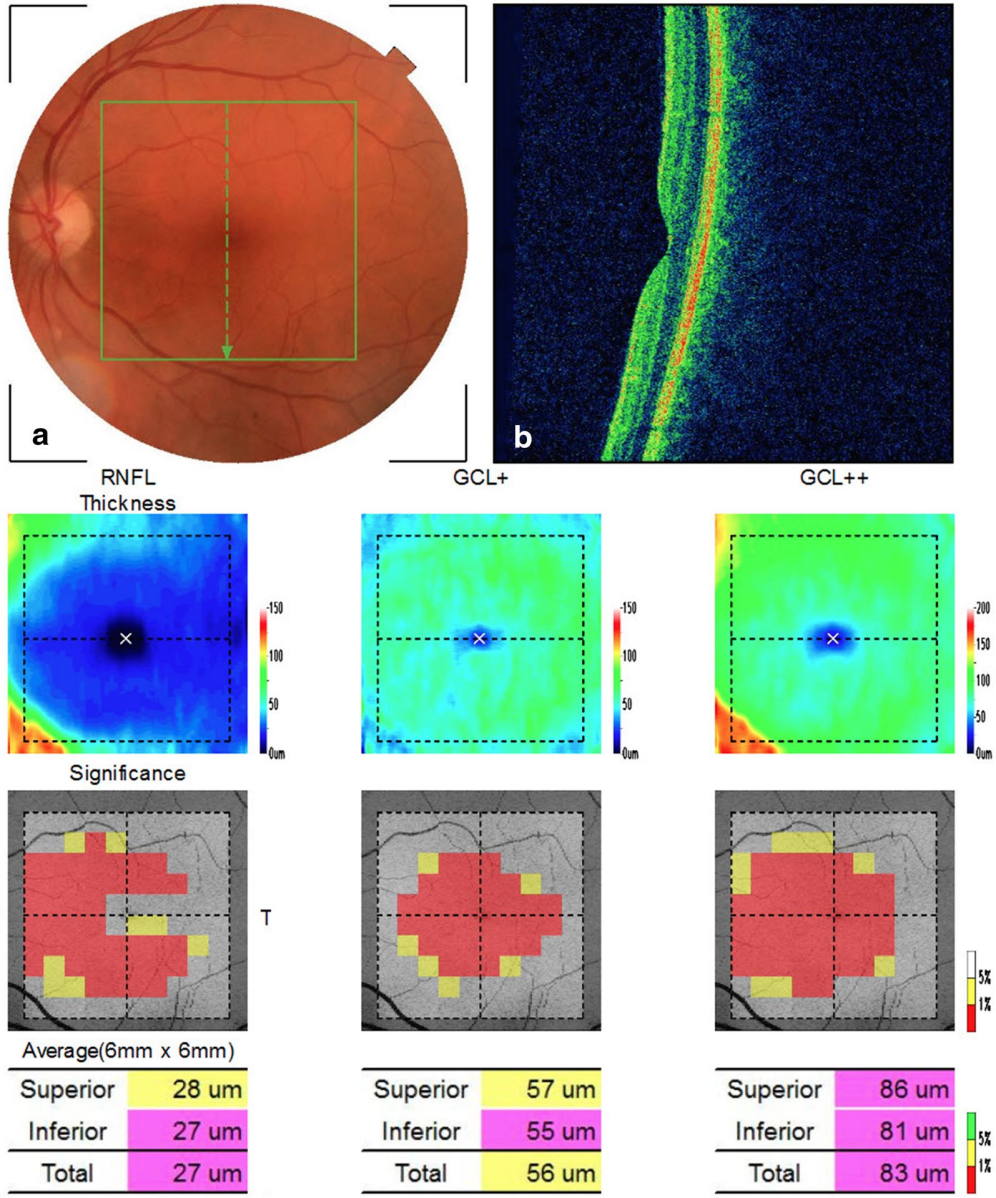
A print out of inner macular analysis using SD-OCT in a patient with Alzheimer disease. The built-in viewer shows the color retinography, oct line scan, macular RNFL thickness (mRNFL), the ganglion cell layer + inner plexiform layer (GCL+) thickness, and the RNFL + GCL + IPL (GCL++) thickness. **a** Fundus color. The *green square line* demarcates the macular area scanned (7 × 7 mm) by the FD-OCT. **b** Optical coherence tomography (*vertical* scan) of macular area. *Center* pseudo-colored map of the measured thickness. *Lower* each grid in the 10 × 10 grid was color coded with no color (within the normal limit), *yellow* (outside of the 95 % normal limit), or *red* (outside of the 99 % normal limit)

In a recently published study [24], we have evaluated 45 eyes of 24 patients with AD using SD-OCT. Our results showed that the macula full-thickness measurements were significantly reduced in all segments, except in the inferior outer segment. The inner segments, around the fovea, were the most affected (Figs. 1, 2). This was an interesting finding and possibly indicates a pattern of neuronal loss in patients with AD. In agreement with our findings, Blank et al., in a histologically analysis, observed a total decrease of one quarter of neurons in GCL at the level of the fovea and parafoveal area of the retina in AD patients [9].

### Inner retinal layers in Alzheimer’s disease patients

In the last decade, the spectral OCT technology evolved significantly allowing for creating high-resolution cross-sectional images. The acquisition speed in SD-OCT reduced the time to acquire three-dimensional images with higher resolution, enhancing the ultrastructural macula analysis, enabling the assessment not only of full macular thickness measurements but also of inner retinal layers. These layers are of particular interest in many ocular and neurological diseases and recent studies based on OCT measurements have demonstrated that the presence of RGC loss may be an early indicator of neural loss in many conditions, such as glaucoma, papilledema, compressive optic neuropathy, multiple sclerosis and optic neuromyelitis [55–58]. This approach may be promising in AD evaluation, especially because the RGC impairment shares similarities with neuronal loss in the brains of these patients. In fact, the reduction of full macular thickness, as shown in previous studies, is possibly related to the preferential involvement of the ganglion cell layer (GCL) [25, 26, 33, 45].

**Fig. 3:**
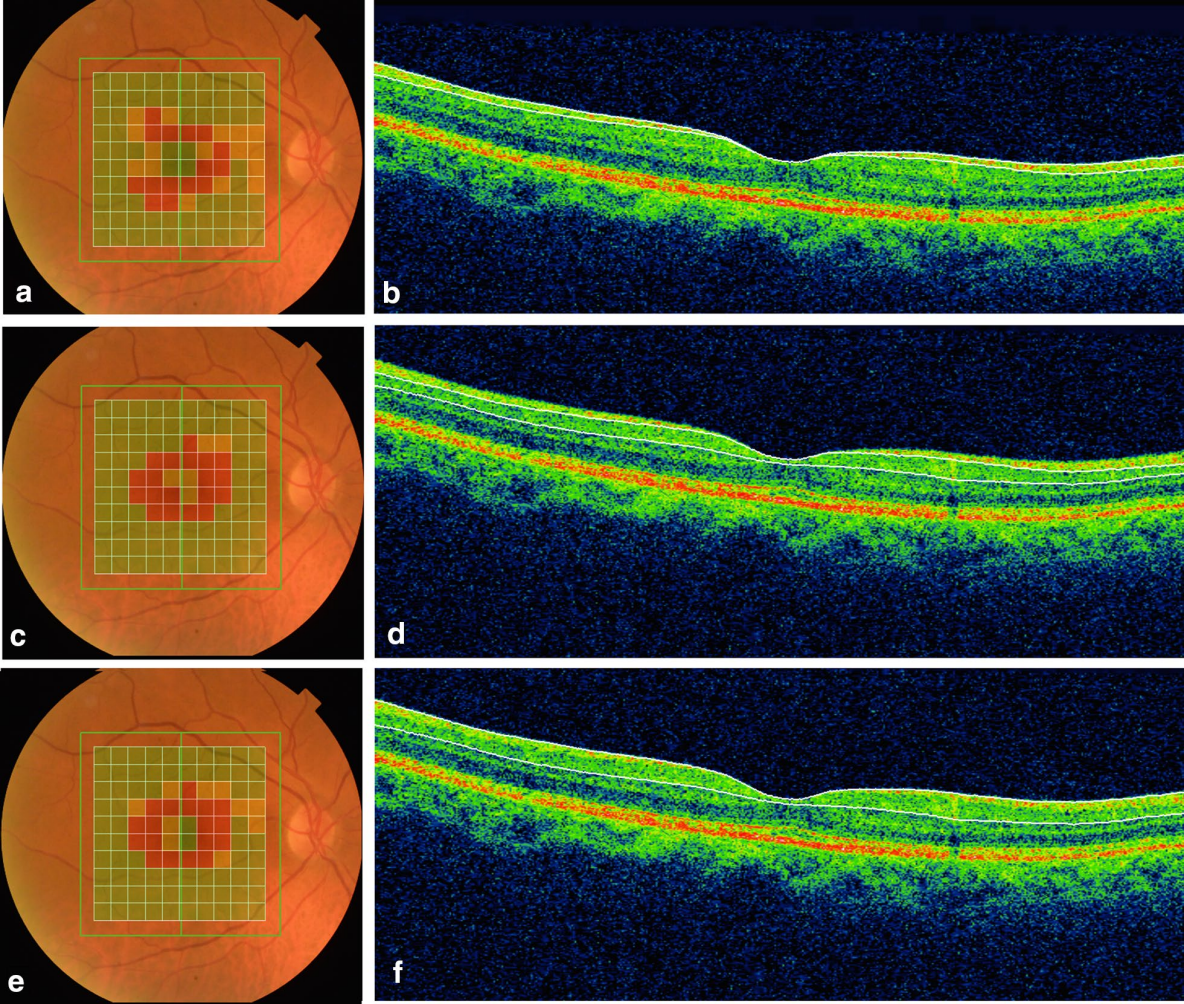
Example of inner macular thickness measurements by SD-OCT of AD patient. On the *left* (**a**, **c**, **e**) represents the scanned area (7 × 7 mm). Each 10 × 10 grid was color coded with no color (within the normal limit), *yellow* (outside the 95 % normal limit), or *red* (outside the 99 % normal limit). The total analyzed area corresponding to 6 × 6 mm. On the *right* vertical OCT scans through the fovea. The *white lines* correspond to the boundaries of the inner retinal layers identified during the segmentation process. **b** Macular RNFL (mRNFL) thickness measurement, through the internal limiting membrane (ILM) to inner boundary of ganglion cell layer (GCL). **d** GCL plus inner plexiform layer (IPL), through the inner boundary of GCL to outer boundary of IPL (GCL+). **f** mRNFL plus GCL plus IPL, trough the ILM to outer boundary of IPL (GCL++)

In 2013, Marziani et al. [31] using SD-OCT, demonstrated a reduction of RNFL + GCL thickness in the nine ETDRS subfields map in AD patients compared with age-matched controls. With the same SD-OCT, another group [43] demonstrated that the average, superior and inferior GCL thickness of the AD patients were significantly thinner than those of the controls. Cheung et al. [59] showed a significantly reduced macular ganglion cell-inner plexiform layer thicknesses in all macular sectors in AD patients compared with cognitively normal controls. In our study [24], the inner macular layers thickness measurements were automatically registered in a square pattern (6 × 6 mm). In each B-scan, the boundaries between the anatomical inner retinal layers in macular area were automatically delimited by built-in software (Fig. 3). The parameters registered in this study were: the average of macular retinal nerve fiber layer (mRNFL) thickness, the average of GCL plus the inner plexiform layer (IPL) thickness, referred as GCL+ and the average of RNFL plus GCL+ thickness, referred as GCL++ (Fig. 3). A pseudo-color thickness map of these layers is shown in Figs. 2 and 3. Our results demonstrate that the GCL+ and GCL++ thickness were significantly lower in eyes of AD patients. The mRNFL thickness measurements in AD eyes were lower than in control eyes, but did not reach statistical significance. Our results demonstrated that inner retinal layers impairment reflect the neuronal degeneration of the retina in AD patients, predominantly affecting the central macular area.

So, as previously demonstrated, the inner retinal layers seem to be preferentially affected in AD. However, information about the integrity of the other retinal layers is crucial. Recent advances in the SD-OCT technology, particularly with improvements in segmentation software analysis, may allow the measurement of all retinal layers. In a recently published study, Garcia-Martin et al. [51], using an automatic segmentation prototype, showed that not only inner retinal layers were reduced in AD patients, but the outer nuclear layers were also impaired. Studies are needed in order to verify the clinical significance of this finding in AD patients and if it’s an indicative of a diffuse degenerative process affecting all retinal layers or as result of retrograde transinaptic degeneration.

## Correlation between RNFL and macular thickness measurements with the cognitive impairment in Alzheimer’s disease patients

Some of the crucial approaches for diagnosis of AD are based on neuropsychological tests such as the mini-mental state examination (MMSE) scores, which are widely used to evaluate the cognitive impairments in dementia patients [2]. So, since the measurement of the degree of severity of cognitive impairment can be given by MMSE score, it seems reasonable that the correlation between this psychological test and OCT parameters could be useful information for clinical evaluation and monitoring of these patients.

In fact, some previous studies have evaluated the correlation between OCT and MMSE score, but the results are somewhat conflicting. While some authors failed to demonstrate a significant correlation between the OCT parameters and MMSE scores [25, 27, 45], others have shown opposite results. Iseri et al. [26] were the first to demonstrate a significant correlation between OCT parameters and MMSE scores in AD patients. However, the correlation was significant only for the total macular volume, and not for the peripapillary RNFL thickness measurements. On the other hand, Ascaso et al. [35] showed a significant association between RNFL thickness in superior and inferior quadrants, and MMSE score. Oktem et al. [42] also demonstrated a significant correlation between MMSE scores and the RNFL parameters. Recently, our group confirmed a significant correlation between MMSE scores and several SD-OCT parameters [24]. For peripapillary RNFL parameters, a significant correlation was found for the average, superior and inferior thickness. For the macular thickness, all parameters (except the superior and inferior outer segments) showed significant correlation with MMSE scores. The most significant correlations were those of the four (superior, inferior, nasal and temporal) inner macular segments and GCL++, reflecting the most affected parameters in our patients. In accordance to our results, Bayhan et al. [43] also demonstrated a significant correlation between MMSE scores and macular GCL thickness. The correlation between cognitive impairment and OCT parameters seems to be stronger for both full macular thickness and inner retinal layers. This is in agreement with the *post mortem* histological study in eyes of AD patients which demonstrated a predominantly RGC loss at the level of the fovea and parafoveal retina [9]. Therefore, macular thickness assessment using OCT, especially with the advances in high-resolution SD-OCT equipments, seems to be a promising diagnostic tool in AD patients. However, further studies, including earlier stages of the disease and monitoring OCT parameters changes along the course of the disease, are required.

## Optical coherence tomography parameters in mild cognitive impairment

The MCI is a clinical condition in which the individual presents with memory loss, larger than expected for their age, but not enough to impair their daily activities [60]. The MCI can be considered as an intermediate stage between normal aging and dementia [61]. The amnestic MCI (aMCI), a subtype with a predominant memory impairment, can be considered by many authors as an early stage of AD, mainly because the rate of progression to Alzheimer in aMCI is 10–15 % per year, while in normal individuals, the conversion rate is only 1–2 % per year [61, 62]. Since MCI may represent an earlier stage of dementia, before the onset of the AD, it is important that these patients undergo careful medical evaluation over the years. Monitoring the clinical status and using ancillary objective tests that could document possible progression to AD are also required. In this scenario, the RNFL thickness and macular measurements by OCT may turn out to represent a non-invasive in vivo biological marker in both MCI and AD patients. Paquet et al. [34] demonstrated that the mean peripapilary RNFL were reduced in both MCI and AD patients, but no significant differences were found between MCI and AD parameters. Similar results were also demonstrated by Oktem et al. [42]. On the other hand, another group found that the total RNFL thickness of MCI patients was significantly different of both AD (thinner) and controls (thicker). The peripapillary RNFL loss was more pronounced in inferior quadrant [27]. Ascaso et al. [35] evaluated peripapillary RNFL thickness and both macular thickness and volume in MCI patients by OCT, and also confirmed a reduction of mean RNFL thickness measurements and in all except from the nasal quadrant when compared to controls. Moreover, these authors found an apparently conflicting data, an increase in macular thickness parameters of MCI patients. They attributed this finding to possible inflammation and/or gliosis that would occur in the early stages of AD.

In a recently published study using SD-OCT, Gao et al. [25] found that the average thickness of the RNFL was reduced in MCI patients compared to AD patients (at inferior quadrant and segments of 5 and 6 o’clock). Compared to controls, MCI patients showed a significant reduction in RNFL thickness measurements only in the temporal quadrant and segments of 8, 9 and 10 o’clock. They also found significant reduction of the macular volume in MCI patients. Other authors showed that the RNFL thickness measurements were reduced in the superior quadrant and the total mean values are gradually and significantly decreased in patients ranging from MCI to severe AD, when compared to the controls [41]. In a systematic review and meta-analysis, Thomson et al. [39] identified five studies including 214 MCI eyes and 421 control eyes), demonstrating a significant reduction in the overall mean RNFL thickness and in all four quadrants (superior, nasal, temporal and inferior) in patients with MCI.

Therefore, as previously demonstrated, peripapillary RNFL and macular thickness parameters in MCI patients were significantly reduced when compared to normal controls. These measurements assessed by OCT demonstrate that axonal loss in MCI is situated in an intermediate stage between AD patients and healthy controls, suggesting the involvement of the retina in early phases even before the onset of dementia.

## Conclusions

In summary, in this article we discussed several clinical applications of OCT in patients with AD. The peripapillary RNFL thickness measurements were reduced in all quadrants, suggesting that a diffuse axonal degeneration occurs in AD patients. This finding is reinforced by the macular thickness reduction, especially by the loss of inner retinal layers, which reflects a preferential retinal GCL impairment in patients with AD. Therefore, OCT parameters can be used to distinguish AD patients from normal aging. Both peripapillary and macular thickness measurements obtained by OCT can be used to detect early neuronal loss as demonstrated in MCI patients, suggesting that OCT could be a promising diagnostic tool in demential diseases. Future studies showing that OCT can be useful in identify the converting patients from MCI to AD are required. Moreover, neuronal loss seems to correlate well with cognitive impairment in AD, especially for macular parameters. This indicates the promising role of OCT in the clinical evaluation of these patients. Therefore, OCT is a non-invasive test, which we believe will serve as a biomarker in AD patients that could be routinely used to evaluate and follow these patients, allowing a more comprehensive approach in this disease.

## References

[1] Blennow K, de Leon MJ, Zetterberg H (2006). Alzheimer’s disease. Lancet.

[2] McKhann G, Drachman D, Folstein M, Katzman R, Price D, Stadlan EM (1984). Clinical diagnosis of Alzheimer’s disease: report of the NINCDS-ADRDA Work Group under the auspices of Department of Health and Human Services Task Force on Alzheimer’s disease. Neurology.

[3] Cronin-Golomb A, Corkin S, Rizzo JF, Cohen J, Growdon JH, Banks KS (1991). Visual dysfunction in Alzheimer’s disease: relation to normal aging. Ann Neurol.

[4] Cronin-Golomb A (1995). Vision in Alzheimer’s disease. Gerontologist.

[5] Armstrong RA (1996). Visual field defects in Alzheimer’s disease patients may reflect differential pathology in the primary visual cortex. Optom Vis Sci.

[6] Morrison JH, Hof PR, Bouras C (1991). An anatomic substrate for visual disconnection in Alzheimer’s disease. Ann NY Acad Sci.

[7] Ferreira LK, Busatto GF (2011). Neuroimaging in Alzheimer’s disease: current role in clinical practice and potential future applications. Clinics.

[8] Blanks JC, Schmidt SY, Torigoe Y, Porrello KV, Hinton DR, Blanks RH (1996). Retinal pathology in Alzheimer’s disease. II. Regional neuron loss and glial changes in GCL. Neurobiol Aging.

[9] Blanks JC, Torigoe Y, Hinton DR, Blanks RH (1996). Retinal pathology in Alzheimer’s disease. I. Ganglion cell loss in foveal/parafoveal retina. Neurobiol Aging.

[10] Hinton DR, Sadun AA, Blanks JC, Miller CA (1986). Optic-nerve degeneration in Alzheimer’s disease. N Engl J Med.

[11] Sadun AA, Bassi CJ (1990). Optic nerve damage in Alzheimer’s disease. Ophthalmology.

[12] Cohen RM, Rezai-Zadeh K, Weitz TM, Rentsendorj A, Gate D, Spivak I (2013). A transgenic Alzheimer rat with plaques, tau pathology, behavioral impairment, oligomeric abeta, and frank neuronal loss. J Neurosci.

[13] Koronyo-Hamaoui M, Koronyo Y, Ljubimov AV, Miller CA, Ko MK, Black KL (2011). Identification of amyloid plaques in retinas from Alzheimer’s patients and noninvasive in vivo optical imaging of retinal plaques in a mouse model. Neuroimage.

[14] Liu B, Rasool S, Yang Z, Glabe CG, Schreiber SS, Ge J (2009). Amyloid-peptide vaccinations reduce β-amyloid plaques but exacerbate vascular deposition and inflammation in the retina of Alzheimer’s transgenic mice. Am J Pathol.

[15] Ning A, Cui J, To E, Ashe KH, Matsubara J (2008). Amyloid-beta deposits lead to retinal degeneration in a mouse model of Alzheimer disease. Invest Ophthalmol Vis Sci.

[16] Perez SE, Lumayag S, Kovacs B, Mufson EJ, Xu S (2009). Beta-amyloid deposition and functional impairment in the retina of the APPswe/PS1DeltaE9 transgenic mouse model of Alzheimer’s disease. Invest Ophthalmol Vis Sci.

[17] Curcio CA, Drucker DN (1993). Retinal ganglion cells in Alzheimer’s disease and aging. Ann Neurol.

[18] Davies DC, McCoubrie P, McDonald B, Jobst KA (1995). Myelinated axon number in the optic nerve is unaffected by Alzheimer’s disease. Br J Ophthalmol.

[19] Parisi V, Restuccia R, Fattapposta F, Mina C, Bucci MG, Pierelli F (2001). Morphological and functional retinal impairment in Alzheimer’s disease patients. Clin Neurophysiol.

[20] Monteiro ML, Cunha LP, Costa-Cunha LV, Maia OO, Oyamada MK (2009). Relationship between optical coherence tomography, pattern electroretinogram and automated perimetry in eyes with temporal hemianopia from chiasmal compression. Invest Ophthalmol Vis Sci.

[21] Monteiro ML, Fernandes DB, Apostolos-Pereira SL, Callegaro D (2012). Quantification of retinal neural loss in patients with neuromyelitis optica and multiple sclerosis with or without optic neuritis using Fourier-domain optical coherence tomography. Invest Ophthalmol Vis Sci.

[22] Monteiro ML, Afonso CL (2014). Macular thickness measurements with frequency domain-OCT for quantification of axonal loss in chronic papilledema from pseudotumor cerebri syndrome. Eye.

[23] Hedges TR, Perez Galves R, Speigelman D, Barbas NR, Peli E, Yardley CJ (1996). Retinal nerve fiber layer abnormalities in Alzheimer’s disease. Acta Ophthalmol Scand.

[24] Cunha LP, Lopes LC, Costa-Cunha LV, Costa CF, Pires LA, Almeida AL (2016). Macular thickness measurements with frequency domain-OCT for quantification of retinal neural loss and its correlation with cognitive impairment in Alzheimer’s disease. PLoS One.

[25] Gao L, Liu Y, Li X, Bai Q, Liu P (2015). Abnormal retinal nerve fiber layer thickness and macula lutea in patients with mild cognitive impairment and Alzheimer’s disease. Arch Gerontol Geriatr.

[26] Iseri PK, Altinas O, Tokay T, Yuksel N (2006). Relationship between cognitive impairment and retinal morphological and visual functional abnormalities in Alzheimer disease. J Neuroophthalmol.

[27] Kesler A, Vakhapova V, Korczyn AD, Naftaliev E, Neudorfer M (2011). Retinal thickness in patients with mild cognitive impairment and Alzheimer’s disease. Clin Neurol Neurosurg.

[28] Kirbas S, Turkyilmaz K, Anlar O, Tufekci A, Durmus M (2013). Retinal nerve fiber layer thickness in patients with Alzheimer disease. J Neuroophthalmol.

[29] Kromer R, Serbecic N, Hausner L, Froelich L, Aboul-Enein F, Beutelspacher SC (2014). Detection of retinal nerve fiber layer defects in Alzheimer’s disease using SD-OCT. Front Psychiatry.

[30] Lu Y, Li Z, Zhang X, Ming B, Jia J, Wang R (2010). Retinal nerve fiber layer structure abnormalities in early Alzheimer’s disease: evidence in optical coherence tomography. Neurosci Lett.

[31] Marziani E, Pomati S, Ramolfo P, Cigada M, Giani A, Mariani C (2013). Evaluation of retinal nerve fiber layer and ganglion cell layer thickness in Alzheimer’s disease using spectral-domain optical coherence tomography. Invest Ophthalmol Vis Sci.

[32] Moreno-Ramos T, Benito-Leon J, Villarejo A, Bermejo-Pareja F (2013). Retinal nerve fiber layer thinning in dementia associated with Parkinson’s disease, dementia with Lewy bodies, and Alzheimer’s disease. J Alzheimers Dis.

[33] Moschos MM, Markopoulos I, Chatziralli I, Rouvas A, Papageorgiou SG, Ladas I (2012). Structural and functional impairment of the retina and optic nerve in Alzheimer’s disease. Curr Alzheimer Res.

[34] Paquet C, Boissonnot M, Roger F, Dighiero P, Gil R, Hugon J (2007). Abnormal retinal thickness in patients with mild cognitive impairment and Alzheimer’s disease. Neurosci Lett.

[35] Ascaso FJ, Cruz N, Modrego PJ, Lopez-Anton R, Santabarbara J, Pascual LF (2014). Retinal alterations in mild cognitive impairment and Alzheimer’s disease: an optical coherence tomography study. J Neurol.

[36] Parisi V (2003). Correlation between morphological and functional retinal impairment in patients affected by ocular hypertension, glaucoma, demyelinating optic neuritis and Alzheimer’s disease. Semin Ophthalmol.

[37] Coppola G, Di Renzo A, Ziccardi L, Martelli F, Fadda A, Manni G (2015). Optical coherence tomography in Alzheimer’s disease: a meta-analysis. PLoS One.

[38] He XF, Liu YT, Peng C, Zhang F, Zhuang S, Zhang JS (2012). Optical coherence tomography assessed retinal nerve fiber layer thickness in patients with Alzheimer’s disease: a meta-analysis. Int J Ophthalmol.

[39] Thomson KL, Yeo JM, Waddell B, Cameron JR, Pal S (2015). A systematic review and meta-analysis of retinal nerve fiber layer change in dementia, using optical coherence tomography. Alzheimers Dement.

[40] Choi SH, Park SJ, Kim NR (2016). Macular ganglion cell-inner plexiform layer thickness is associated with clinical progression in mild cognitive impairment and Alzheimers disease. PLoS One.

[41] Liu D, Zhang L, Li Z, Zhang X, Wu Y, Yang H (2015). Thinner changes of the retinal nerve fiber layer in patients with mild cognitive impairment and Alzheimer’s disease. BMC Neurol.

[42] Oktem EO, Derle E, Kibaroglu S, Oktem C, Akkoyun I, Can U (2015). The relationship between the degree of cognitive impairment and retinal nerve fiber layer thickness. Neurol Sci.

[43] Bayhan HA, Aslan Bayhan S, Celikbilek A, Tanik N, Gurdal C (2015). Evaluation of the chorioretinal thickness changes in Alzheimer’s disease using spectral-domain optical coherence tomography. Clin Exp Ophthalmol.

[44] Garcia-Martin ES, Rojas B, Ramirez AI, de Hoz R, Salazar JJ, Yubero R (2014). Macular thickness as a potential biomarker of mild Alzheimer’s disease. Ophthalmology.

[45] Polo V, Garcia-Martin E, Bambo MP, Pinilla J, Larrosa JM, Satue M (2014). Reliability and validity of cirrus and spectralis optical coherence tomography for detecting retinal atrophy in Alzheimer’s disease. Eye.

[46] Berisha F, Feke GT, Trempe CL, McMeel JW, Schepens CL (2007). Retinal abnormalities in early Alzheimer’s disease. Invest Ophthalmol Vis Sci.

[47] Larrosa JM, Garcia-Martin E, Bambo MP, Pinilla J, Polo V, Otin S (2014). Potential new diagnostic tool for Alzheimer’s disease using a linear discriminant function for Fourier domain optical coherence tomography. Invest Ophthalmol Vis Sci.

[48] Bambo MP, Garcia-Martin E, Gutierrez-Ruiz F, Pinilla J, Perez-Olivan S, Larrosa JM (2015). Analysis of optic disk color changes in Alzheimer’s disease: a potential new biomarker. Clin Neurol Neurosurg.

[49] Oktem EO, Derle E, Kibaroglu S, Oktem C, Akkoyun I, Can U (2015). The relationship between the degree of cognitive impairment and retinal nerve fiber layer thickness. Neurol Sci..

[50] Salobrar-Garcia E, Hoyas I, Leal M, de Hoz R, Rojas B, Ramirez AI (2015). Analysis of retinal peripapillary segmentation in early Alzheimer’s disease patients. Biomed Res Int.

[51] Garcia-Martin E, Bambo MP, Marques ML, Satue M, Otin S, Larrosa JM (2016). Ganglion cell layer measurements correlate with disease severity in patients with Alzheimer’s disease. Acta Ophthalmol..

[52] Costa-Cunha LV, Cunha LP, Malta RF, Monteiro ML (2009). Comparison of Fourier-domain and time-domain optical coherence tomography in the detection of band atrophy of the optic nerve. Am J Ophthalmol.

[53] Wojtkowski M, Bajraszewski T, Gorczynska I, Targowski P, Kowalczyk A, Wasilewski W (2004). Ophthalmic imaging by spectral optical coherence tomography. Am J Ophthalmol.

[54] Wojtkowski M, Srinivasan V, Ko T, Fujimoto J, Kowalczyk A, Duker J (2004). Ultrahigh-resolution, high-speed, Fourier domain optical coherence tomography and methods for dispersion compensation. Opt Express.

[55] Afonso CL, Raza AS, Kreuz AC, Hokazono K, Cunha LP, Oyamada MK (2015). Relationship between pattern electroretinogram, frequency-domain OCT, and automated perimetry in chronic papilledema from pseudotumor cerebri syndrome. Invest Ophthalmol Vis Sci.

[56] Fernandes DB, Raza AS, Nogueira RG, Wang D, Callegaro D, Hood DC (2013). Evaluation of inner retinal layers in patients with multiple sclerosis or neuromyelitis optica using optical coherence tomography. Ophthalmology.

[57] Hood DC, Slobodnick A, Raza AS, de Moraes CG, Teng CC, Ritch R (2014). Early glaucoma involves both deep local, and shallow widespread, retinal nerve fiber damage of the macular region. Invest Ophthalmol Vis Sci.

[58] Monteiro ML, Hokazono K, Fernandes DB, Costa-Cunha LV, Sousa RM, Raza AS (2014). Evaluation of inner retinal layers in eyes with temporal hemianopic visual loss from chiasmal compression using optical coherence tomography. Invest Ophthalmol Vis Sci.

[59] Cheung CY, Ong YT, Hilal S, Ikram MK, Low S, Ong YL (2015). Retinal ganglion cell analysis using high-definition optical coherence tomography in patients with mild cognitive impairment and Alzheimer’s disease. J Alzheimers Dis.

[60] Petersen RC, Smith GE, Waring SC, Ivnik RJ, Tangalos EG, Kokmen E (1999). Mild cognitive impairment: clinical characterization and outcome. Arch Neurol.

[61] Grundman M, Petersen RC, Ferris SH, Thomas RG, Aisen PS, Bennett DA (2004). Mild cognitive impairment can be distinguished from Alzheimer disease and normal aging for clinical trials. Arch Neurol.

[62] Petersen RC, Stevens JC, Ganguli M, Tangalos EG, Cummings JL, DeKosky ST (2001). Practice parameter: early detection of dementia: mild cognitive impairment (an evidence-based review). Report of the Quality Standards Subcommittee of the American Academy of Neurology. Neurology.

